# Discordance Between Triglycerides, Remnant Cholesterol and Systemic Inflammation in Patients with Schizophrenia

**DOI:** 10.3390/biomedicines12122884

**Published:** 2024-12-18

**Authors:** Jeffrey Wang, Maaike Kockx, Gabrielle J. Pennings, Tim Lambert, Vincent Chow, Leonard Kritharides

**Affiliations:** 1 Atherosclerosis and Vascular Biology Laboratory, The ANZAC Research Institute, Concord Repatriation General Hospital, University of Sydney, Concord 2138, Australia; jeffrey.wang1@sydney.edu.au (J.W.); gabrielle.pennings@sydney.edu.au (G.J.P.); leonard.kritharides@sydney.edu.au (L.K.); 2 Concord Clinical School, Faculty of Medicine and Health, University of Sydney, Camperdown 2050, Australia; tim.lambert@sydney.edu.au (T.L.); vincent.chow@sydney.edu.au (V.C.); 3 Collaborative Centre for Cardiometabolic Health, Charles Perkins Centre, University of Sydney, Camperdown 2050, Australia; 4 Department of Cardiology, Concord Repatriation General Hospital, Sydney Local Health District, Concord 2138, Australia

**Keywords:** schizophrenia, hypertriglyceridaemia, remnant cholesterol, systemic inflammation

## Abstract

Background/Objectives: Hypertriglyceridaemia and systemic inflammation are prevalent in patients with schizophrenia and contribute to an increased risk of cardiovascular disease. Although elevated triglycerides (TGs) and remnant cholesterol are linked to inflammation in the general population and individuals with metabolic syndrome, whether they are associated in patients with schizophrenia remains unclear. Methods: Fasting levels of TG, cholesterol (total cholesterol (TC), low-density lipoprotein cholesterol (LDL-C), high-density lipoprotein cholesterol (HDL-C) and remnant cholesterol)), and markers of systemic inflammation including high-sensitivity C-reactive protein (hsCRP), leukocyte counts and their differentials (neutrophils, monocytes and lymphocytes) were determined in 147 patients diagnosed with schizophrenia on long-term antipsychotic regimens and compared with 56 age- and sex-matched healthy controls. Apolipoprotein B and glycosylation of acute phase reactant (GlycA) signatures were assessed by NMR. Circulating cytokine levels were measured by a cytokine/chemokine multiplex assay. Results: Patients with schizophrenia had markedly elevated TG and remnant cholesterol relative to controls and had evidence of systemic inflammation with increased circulating hsCRP, GlycA, leukocyte, neutrophil counts and neutrophil-to-lymphocyte ratio (NLR). Unexpectedly TG and remnant cholesterol did not correlate with systemic inflammatory markers in patients with schizophrenia, and differences in inflammatory markers between controls and patients persisted after adjusting for the lipid profile. Interleukin (IL)-10 levels were increased in patients with schizophrenia, suggesting an anti-inflammatory signature. Conclusions: The discordance between TG, remnant cholesterol and systemic inflammation in patients with schizophrenia suggests these are likely independent contributors to cardiovascular risk in this population.

## 1. Introduction

Patients with schizophrenia (SZ) have an increased risk of cardiovascular disease (CVD) compared to individuals from the general population [1]. This is associated with an increased prevalence of obesity, diabetes and dyslipidaemia, with dyslipidaemia decreasing overall survival rates by 10% over 10 years in this population [2]. Patients often display a metabolic syndrome-type phenotype with increased body mass index (BMI), fasting blood glucose, blood pressure and triglycerides (TGs) and decreased high-density lipoprotein cholesterol (HDL-C) [3]. In particular, elevated plasma TG is recognised as an established risk factor for incident CVD independent of elevated low-density lipoprotein cholesterol (LDL-C) levels [4] and is associated with increased remnant cholesterol and apolipoprotein (apo) CIII concentrations in SZ [5].

Inflammation plays a critical role in the pathophysiology of CVD [6], with systemic inflammation significantly contributing to premature mortality in patients with SZ with an odds ratio of 1.19 [7]. Different markers of inflammation provide insights into distinct aspects of the inflammatory response in chronic conditions and can differ according to the populations and clinical conditions studied. While the acute phase reactant C-reactive protein (CRP) is often reported [8], cellular markers such as the leukocyte count and neutrophil-to-lymphocyte ratio (NLR), pro- and anti-inflammatory cytokines and the more recently characterised GlycA, a composite marker derived from nuclear magnetic resonance (NMR), have all been associated with increased risk of cardiovascular events [9,10,11].

Mendelian randomisation studies have indicated that high remnant cholesterol levels are causally associated with low-grade systemic inflammation, evidenced by elevated high-sensitivity CRP (hsCRP) levels, and increased risk of CVD [12]. Mechanistic studies have observed that remnant cholesterol can induce arterial inflammation and promote foam cell formation, monocyte activation and adhesion and endothelial dysfunction [13]. Additionally, TG and triglyceride-rich lipoprotein (TRL) particles are capable of activating circulating neutrophils and monocytes [14,15].

There is growing evidence that inflammation and the immune response are perturbed and linked to the aetiology of SZ, which may be modified by the use of antipsychotic medications [16,17]. Many studies report low-grade inflammation in SZ and other psychiatric disorders that is characterised by elevated cytokines, acute phase reactants and white blood cell (WBC) counts [18,19,20], and several studies have found these to be associated with components of the metabolic syndrome [19,21,22]. However, the contribution of TG and remnant cholesterol to the systemic inflammation observed in SZ remains unclear. Here, we investigate the relationships between TG and remnant cholesterol with circulating levels of hsCRP and GlycA, leukocyte counts and cytokine levels in a well-characterised cohort of patients with SZ on long-term antipsychotic medication.

## 2. Materials and Methods

### 2.1. Subjects

The patients with SZ and healthy controls included in this study were previously described [5,23,24,25]. The study was performed under the ethical guidelines of the 1975 Declaration of Helsinki, and all subjects were recruited with approval from the Sydney Local Health District Human Research Ethics Committee (HREC/11/CRGH/269). Written informed consent was obtained from all subjects. We have previously published on ventricular dysfunction, hypercoagulability, hypertriglyceridaemia and HDL function in this cohort [23,24,26]. For the current study, 147 patients and 56 age and sex-matched healthy controls for whom inflammation markers were determined were included. Most patients were prescribed clozapine (119). Patients not on clozapine received quetiapine (5), paliperidone (3), risperidone (2), aripiprazole (3), clopixol (2), intra-muscular (2), amisulpride (1), ziprasidone (1) or lithium (2), and three patients were prescribed with multiple antipsychotic medications. None of the healthy controls and 30 of the patients with SZ were receiving lipid-lowering therapy (statin).

### 2.2. Biochemical Analyses

Total cholesterol (TC), HDL-C, LDL-C and TG (where LDL-C was calculated using the Friedewald equation [27]), glycated haemoglobin (HbA1c), neutrophil, monocyte and lymphocyte count and high-sensitivity C-reactive protein (hsCRP) were all determined as described previously [5,23,26]. In healthy controls, WBC counts were available in 22 subjects. The NLR value was calculated by dividing the neutrophil count by the lymphocyte count, while the monocyte-to-lymphocyte ratio (MLR) value was calculated by dividing the monocyte count by the lymphocyte count. Plasma cytokine (IL-1β, IL-6, TNF-α, IFN-γ, IL-4 and IL-10) concentrations were measured using the MILLIPLEX MAP Human Cytokine/Chemokine panel kit (Abacus) in a subset of healthy controls (n = 19) and patients with SZ (n = 53).

### 2.3. NMR Spectroscopy

ApoB and glycosylation of acute phase reactant (GlycA) signatures (a composite biomarker of the acute phase response [28]) were measured by NMR spectroscopy on a Vantera Clinical Analyser platform at LabCorp (Morrisville, NC, USA) as previously described [29].

### 2.4. Statistical Analysis

Data are presented as the mean ± standard deviation (SD) (normal distribution) or median (IQR) (not normally distributed variables). Categorical variables are presented as frequencies (%). Comparisons between healthy controls and patients with SZ were investigated using an unpaired *t*-test (parametric distributions) or Mann–Whitney U test (nonparametric distributions) and adjusted using linear regression models, with Bonferroni adjustment for multiple comparisons. Not normally distributed variables were logarithm base 10 (log_10_) transformed. *p* < 0.05 was considered statistically significant. Analyses were performed using Graphpad Prism 10, or IBM SPSS Statistics (version 28).

## 3. Results

The clinical characteristics of healthy controls and patients with SZ in this study are reported in Table 1 and differed in BMI, smoking history, history of diabetes and statin therapy.

In the unadjusted analysis, patients with SZ had markedly higher plasma TG and remnant cholesterol and lower HDL-C levels compared to controls, but similar TC, LDL-C and apoB levels (Table 2), and this was observed in both men and women with schizophrenia (Supplementary Table S1). After adjusting for age, sex, BMI, diabetes, smoking and statin use, plasma HDL-C, TG and remnant cholesterol remained significantly different between controls and patients with SZ (Table 2).

Patients with SZ showed increased levels of both circulating hsCRP and GlycA compared with controls (both *p* < 0.001) and this was observed in both men and women with schizophrenia (Supplementary Table S2). Both hsCRP and GlycA remained significant after adjustment for age, sex, BMI, diabetes, smoking and statin use (*p* < 0.001, Table 3).

Patients also showed increased WBC count, neutrophil count, NLR and MLR in the unadjusted analysis (Table 4). After adjustment for clinical variables, WBC was no longer significant, neutrophil and lymphocyte counts were of borderline statistical significance and only NLR remained significantly different between patients and controls (*p* = 0.005).

Our analysis of patients with SZ according to different antipsychotic agents, specifically clozapine vs. non-clozapine use, revealed no difference in lipid parameters by treatment type; however lymphocyte count (1.9 [1.5–2.5 × 10^9^/L] vs. 2.2 [1.9–2.6]; *p* = 0.03) and NLR (2.5 [1.9–3.4 × 10^9^/L] vs. 2.0 [1.6–2.5]; *p* = 0.01) were higher in patients receiving clozapine compared to those not receiving clozapine.

We next investigated the relationships between TG and remnant cholesterol levels and inflammatory markers in the cohort with SZ. Unexpectedly, TG and remnant cholesterol levels did not correlate with circulating hsCRP, GlycA, total WBC, neutrophil count, NLR or MLR (Table 5). Similar findings were observed for apoB and HDL-C levels (Table 5).

To investigate whether differences in plasma TG and remnant cholesterol levels between controls and patients with SZ were explained by systemic inflammation, a general linear modelling analysis was undertaken. TG and remnant cholesterol levels remained significantly higher, while HDL-C levels remained significantly lower in patients with SZ after adjusting for hsCRP, GlycA or both (Table 6).

We also investigated whether differences in hsCRP and GlycA levels could be explained by TG or remnant cholesterol levels and confirmed that they were not as they remained significantly higher in patients with SZ compared to controls after adjusting for TG or remnant cholesterol, as well as HDL-C or apoB (Table 7).

To explore potential cytokine mediators of the systemic inflammatory response in patients with SZ, we analysed cytokine levels in a subset of healthy controls (n = 19) and patients (n = 53). Clinical characteristics (Supplementary Table S3), lipid parameters (Supplementary Table S4) and systemic inflammatory markers (Supplementary Table S5) were similar between this subset cohort and the whole cohort. In unadjusted analysis, there was a trend for TNF-α to be higher in patients (*p* = 0.05), while IL-10 levels were significantly increased (*p* = 0.004). IL-6/IL-10 and TNF-α/IL-10 ratios, which have been previously reported as indicators of the pro-to-anti-inflammatory balance [30,31], were significantly lower in patients with SZ compared to healthy controls (Table 8). In contrast, IL-1β, IL-6, IL-4, TNF-α and IFN-γ levels and IFN-γ/IL-4 and IFN-γ/IL-10 ratios were similar between controls and patients (Table 8). In our multivariable analysis that adjusted for clinical characteristics, IL-10 levels remained significantly higher, while IL-6/IL-10 and TNF-α/IL-10 ratios remained lower in patients in contrast to controls (Supplementary Table S6).

Our comparison of patients with SZ based on clozapine use revealed that IL-10 levels were higher (8.4 [4.7–22.1 pg/mL] vs. 2.3 [0.7–7.8]; *p* = 0.01), and IL-6/IL-10 (0.9 [0.4–1.6] vs. 2.0 [1.0–4.7]; *p* = 0.01) and TNF-α/IL-10 (1.1 [0.5–2.2] vs. 2.8 [1.3–7.0]; *p* = 0.03) ratios were lower in patients receiving clozapine compared to those not receiving clozapine, suggesting that clozapine promotes an anti-inflammatory cytokine profile.

TG (Spearman’s rho −0.31, *p* = 0.02) and remnant cholesterol (Spearman’s rho −0.34, *p* = 0.02) levels were negatively and only moderately correlated with IL-6 levels but not correlated with other cytokines (Table 9). ApoB levels were negatively and moderately correlated with IL-1β, IL-6 and IL-4 levels, while HDL-C levels were negatively and moderately correlated with IFN-γ and IL-4 levels (Table 9).

## 4. Discussion

In the present study, we examined the relationship between TG and remnant cholesterol with biomarkers of low-grade, systemic inflammation in patients with chronic SZ on long-term antipsychotics. We found that patients with SZ had elevated TG and remnant cholesterol and evidence of systemic inflammation as demonstrated by increased hsCRP and GlycA, WBC, neutrophil counts and NLR. These changes remained significant after adjusting for clinical characteristics and comorbidities including age, obesity (BMI), diabetes and smoking. Unexpectedly, TG and remnant cholesterol were not correlated with hsCRP and GlycA levels, leukocyte and neutrophil counts nor NLR in patients with SZ. Consistent with these results, TG and remnant cholesterol differences between healthy controls and patients remained significant after adjusting for hsCRP and GlycA, and hsCRP and GlycA differences remained significant after adjusting for TG and remnant cholesterol. Collectively, these results suggest discordance between low-grade inflammation and TG and remnant cholesterol changes in SZ.

Several biomarkers are routinely utilised as indicators of systemic inflammation. High-sensitivity CRP is an acute phase reactant released by hepatocytes in response to cytokine signalling, namely IL-6, and elevated levels directly reflect a systemic inflammatory state and are independently associated with future risk of cardiovascular events [8,32]. GlycA is a novel NMR-derived marker of systemic inflammation that measures the proton spectroscopy signal of methyl groups in N-acetyl glucosamine residues found in many acute phase reactant proteins [11,28,33]. As a composite marker with lower intra-individual variability as opposed to measures of single acute phase candidates such as hsCRP, GlycA is an emerging marker of systemic inflammation that has been associated with CVD independent of traditional risk factors [34,35,36]. To the best of our knowledge, this is the first study to report on GlycA changes in SZ, making our findings novel. On the other hand, cellular markers and cytokines provide more mechanistic insights into pro- and anti-inflammatory responses during systemic inflammation. In particular, neutrophil, lymphocyte counts and NLR reflect dynamic changes in the innate and adaptive immune responses and have all been proposed as independent risk factors in predicting major CVD [9,37,38]. Pro-inflammatory cytokines such as IL-1β, IL-6 and TNF-α and anti-inflammatory cytokines such as IL-4 and IL-10 provide further insights into the mediators of cellular and systemic responses and have been linked to causal pathways related to atherosclerosis and thrombosis [10,39,40,41,42].

Mendelian and observational studies robustly support the association between elevated remnant cholesterol and low-grade, systemic inflammation in the general population and in individuals with metabolic syndrome [12,43,44]. TG and remnant particles have also been associated with markers including leukocytes and GlycA levels [14]. Recent studies have observed that concurrent elevations in both remnant cholesterol and CRP were associated with a higher risk of atherosclerotic CVD than elevations in either of the factors individually, indicating that the combined presence of elevated remnant cholesterol and CRP may further increase CVD risk [45,46]. In our study, although we observed increased TG, remnant cholesterol and systemic inflammatory markers in patients with SZ, they were not correlated, and differences in inflammatory markers persisted after adjusting for lipid levels. These findings indicate that in SZ, there are additional contributors to apparent systemic inflammation other than those attributable to metabolic comorbidities such as hyperlipidaemia.

Inflammation in SZ has been well-characterised. Genetic studies have identified links between immune response genes [47,48], while epidemiological data demonstrate associations between autoimmune disorders and severe infections with increased risk of SZ [49,50]. Evidence from meta-analyses shows that circulating inflammatory biomarker levels, including leukocytes, acute phase reactants, and cytokines, are elevated in patients with SZ, consistent with our findings [17,51,52,53,54]. Although several studies have examined relationships between low-grade, systemic inflammation and metabolic syndrome in SZ, the data on TG appear inconsistent as some report positive associations while others find no associations [19,20,21,22,55]. However, to the best of our knowledge, there have been no reports on the relationship between remnant cholesterol and inflammation in SZ, making our findings novel. The discrepancy in the findings may be attributed to heterogeneous study designs, with differences in sample size, confounding factors and SZ disease stage and treatment setting. In this study, we analysed patients with chronic SZ who were on long-term antipsychotic medication treatment, with the majority of subjects on clozapine therapy, which may represent a distinct subset of the SZ population with different metabolic and inflammatory profiles.

The pleiotropic effects of antipsychotic medications have been reported. There is growing evidence that antipsychotics, in particular atypical drugs, may directly induce or exacerbate genetic predisposition to weight gain, obesity and dyslipidaemia [56,57,58]. Studies in humans have also shown that antipsychotic medications can attenuate pro-inflammatory signatures in SZ, with heterogeneous effects observed between different medications [17,51,59]. Animal and cell studies indicate that antipsychotics such as clozapine can directly promote triglyceride production through the activation of sterol regulatory element-binding protein (SREBP) and may exert an anti-inflammatory effect through the inhibition of NLRP3 activation [60,61]. Therefore, our finding of discordance between TG, remnant cholesterol and inflammation in SZ may be associated with the metabolic and anti-inflammatory impact of antipsychotic treatment.

We observed higher IL-10 levels in patients with SZ in contrast to healthy controls, which may reflect an overall anti-inflammatory balance in response to antipsychotic treatment. IL-10 is known for its potent anti-inflammatory and immunomodulatory functions [62]. It inhibits the production of CRP, IL-6, IL-1β, TNF-α and IFN-γ and inhibits antigen presentation and T-cell responses [62,63]. In the central nervous system, the role of inflammation in the aetiology of SZ has been postulated [17], and as IL-10 is known to limit neuronal damage due to inflammation, it may have an important role in SZ biology. The persisting inflammation in our SZ patients despite higher IL-10 levels suggests that the anti-inflammatory effect of IL-10 is insufficient to control all pro-inflammatory processes. These results would also suggest that the apparent systemic inflammation in SZ is not simply explained by either a definitive increase or decrease in the measured pro- and anti-inflammatory responses, respectively.

Early reports indicate that clozapine is associated with decreases in CRP, IL-1β and IL-6 levels in treatment-resistant patients; however, follow-up results have been inconsistent, and the cytokine-associated effects of clozapine remain unclear [64,65,66]. Clozapine may have distinct effects on the cell-mediated immune response, as clozapine patients had higher NLR compared to those not receiving clozapine, as previously reported [24,67,68]. Although it has been hypothesised that increased granulocyte colony-stimulating factor, TNF-α, IL-2 and IL-6 levels may play a role, the mechanisms have yet to be defined [69]. In our study, the number of patients on antipsychotics other than clozapine was too small to investigate the differences between antipsychotic medications, and as it was not feasible to compare with a naïve, untreated control group, this remains a study limitation.

In conclusion, we have demonstrated that in SZ, although there are concomitant increases in TG, remnant cholesterol and systemic inflammatory markers, they do not appear to be associated. These findings suggest that systemic inflammation may be independent of TG dyslipidaemia and that remnant cholesterol and inflammation may be independent contributors to CVD risk in these patients.

## Figures and Tables

**Table 1:** Clinical characteristics of healthy controls (n = 56) and patients with schizophrenia (n = 147).

	Healthy Controls (n = 56)	SZ (n = 147)	*p* Value
Age, years	38.5 (31.3–49.8)	40.0 (30.0–51.0)	ns
Biological sex, M/F; %M	29/27 (52)	97/50 (66)	ns
BMI, kg/m^2^	24.0 (22.5–26.4)	28.4 (24.5–32.9)	<0.001
HbA1c, %	5.4 (5.3–5.6)	5.7 (5.4–6.0)	0.04
Smoking, % current	2/56 (4)	64/147 (44)	<0.001
Diabetes, %	2/56 (4)	30/147 (20)	0.004
Statin therapy, %	0/56 (0)	30/146 (21)	<0.001
Hypertension, %	4/53 (8)	16/133 (12)	ns
SZ duration of illness, years	-	15.3 (8.8–23.6)	-
Clozapine therapy, %	-	119/147 (81)	-

Results are expressed as number and (%), or median and (interquartile range). HbA1c levels were determined in 22 controls and 136 patients. ns = non-significant; SZ = schizophrenia; BMI = body mass index; HbA1c = haemoglobin A1c.

**Table 2:** Unadjusted and multivariable-adjusted analysis of lipid parameters between healthy controls (n = 56) and patients with SZ (n = 147).

	Healthy Controls (n = 56)	SZ (n = 147)	*p* Value
**Unadjusted**			
TC, mmol/L	4.9 (4.3–5.6)	5.0 (4.2–5.7)	ns
LDL-C mmol/L	2.7 (2.3–3.5)	3.0 (2.3–3.6)	ns
HDL-C, mmol/L	1.4 (1.2–1.8)	1.1 (0.9–1.4)	<0.001
TG, mmol/L	0.9 (0.7–1.5)	1.7 (1.2–2.4)	<0.001
Remnant cholesterol, mmol/L	0.4 (0.3–0.7)	0.8 (0.5–1.1)	<0.001
apoB, µmol/L	1.6 (1.3–2.0)	1.6 (1.4–2.0)	ns
**Multivariable-adjusted**			
Log_10_ TC, mmol/L	0.65 ± 0.02	0.65 ± 0.01	ns
Log_10_ LDL-C, mmol/L	0.31 ± 0.03	0.35 ± 0.02	ns
Log_10_ HDL-C, mmol/L	0.12 ± 0.02	0.05 ± 0.01	0.002
Log_10_ TG, mmol/L	0.08 ± 0.05	0.25 ± 0.03	<0.001
Log_10_ remnant cholesterol, mmol/L	−0.29 ± 0.05	−0.17 ± 0.03	0.004
Log_10_ apoB, µmol/L	0.22 ± 0.03	0.20 ± 0.02	ns

Results are expressed as median and (interquartile range). For multivariable-adjusted analysis, results are expressed as estimated means ± standard error. Multivariable model was adjusted for age, sex, body mass index, diabetes, smoking and statin use. ApoB levels were determined in 46 controls and 104 patients. ns = non-significant; SZ = schizophrenia; TC = total cholesterol; LDL-C = low-density lipoprotein cholesterol; HDL-C = high-density lipoprotein cholesterol; TG = triglyceride; apo = apolipoprotein.

**Table 3:** Unadjusted and multivariable-adjusted analysis of circulating hsCRP and GlycA levels between healthy controls (n = 54) and patients with SZ (n = 147).

	Healthy Controls (n = 54)	SZ (n = 147)	*p* Value
**Unadjusted**			
Log_10_ hsCRP, mg/L	−0.11 ± 0.50	0.37 ± 0.50	<0.001
Log_10_ GlycA, µmol/L	2.56 ± 0.08	2.65 ± 0.08	<0.001
**Adjusted for age and sex**			
Log_10_ hsCRP, mg/L	−0.11 ± 0.07	0.37 ± 0.04	<0.001
Log_10_ GlycA, µmol/L	2.56 ± 0.01	2.65 ± 0.01	<0.001
**Adjusted for age, sex, body mass index, diabetes, smoking and statin use**			
Log_10_ hsCRP, mg/L	−0.01 ± 0.10	0.30 ± 0.06	<0.001
Log_10_ GlycA, µmol/L	2.57 ± 0.02	2.65 ± 0.01	<0.001

For unadjusted analysis, results are expressed as mean ± standard deviation. For adjusted analyses, results are expressed as estimated marginal means ± standard error, and non-parametrically distributed variables were log_10_ transformed before analysis. GlycA levels were determined in 46 controls and 104 patients. log_10_ = logarithm base 10; SZ = schizophrenia; hsCRP = high-sensitivity C-reactive protein.

**Table 4:** Unadjusted and multivariable-adjusted analysis of leukocyte counts between healthy controls (n = 22) and patients with SZ (n = 147).

	Healthy Controls (n = 22)	SZ (n = 147)	*p* Value
**Unadjusted**			
Log_10_ WBC, ×10^9^/L	0.80 ± 0.09	0.88 ± 0.14	0.002
Log_10_ PMN, ×10^9^/L	0.54 ± 0.13	0.68 ± 0.18	<0.001
Log_10_ Monocytes, ×10^9^/L	−0.34 ± 0.16	−0.26 ± 0.16	ns
Log_10_ Lymphocytes, ×10^9^/L	0.32 ± 0.12	0.29 ± 0.15	ns
Log_10_ NLR	0.22 ± 0.18	0.38 ± 0.22	<0.001
Log_10_ MLR	−0.67 ± 0.15	−0.56 ± 0.20	0.008
**Adjusted for age and sex**			
Log_10_ WBC, ×10^9^/L	0.80 ± 0.03	0.88 ± 0.01	0.01
Log_10_ PMN, ×10^9^/L	0.54 ± 0.04	0.68 ± 0.02	<0.001
Log_10_ Monocytes, ×10^9^/L	−0.34 ± 0.04	−0.27 ± 0.02	ns
Log_10_ Lymphocytes, ×10^9^/L	0.32 ± 0.03	0.29 ± 0.01	ns
Log_10_ NLR	0.22 ± 0.05	0.38 ± 0.02	0.001
Log_10_ MLR	−0.68 ± 0.05	−0.56 ± 0.02	0.02
**Adjusted for age, sex, body mass index, diabetes, smoking and statin use**			
Log_10_ WBC, mg/L	0.89 ± 0.03	0.92 ± 0.02	ns
Log_10_ PMN, ×10^9^/L	0.62 ± 0.05	0.71 ± 0.02	0.035
Log_10_ Monocytes, ×10^9^/L	−0.27 ± 0.05	−0.24 ± 0.02	ns
Log_10_ Lymphocytes, ×10^9^/L	0.39 ± 0.04	0.32 ± 0.02	0.044
Log_10_ NLR	0.24 ± 0.06	0.40 ± 0.03	0.005
Log_10_ MLR	−0.66 ± 0.06	−0.57 ± 0.03	ns

For unadjusted analysis, results are expressed as mean ± standard deviation. For adjusted analyses, results are estimated marginal means ± standard error. log_10_ = logarithm base 10; ns = non-significant; SZ = schizophrenia; WBC = white blood cell; PMN = polymorphonuclear neutrophil; NLR = neutrophil-to-lymphocyte ratio; MLR = monocyte-to-lymphocyte ratio.

**Table 5:** Spearman’s rank correlations between lipids and circulating inflammatory markers in patients with schizophrenia (n = 147).

	HDL-C, mmol/L		TG, mmol/L		Remnant Cholesterol, mmol/L		apoB, µmol/L	
	ρ	*p* Value	ρ	*p* Value	ρ	*p* Value	ρ	*p* Value
hsCRP, mg/L	−0.14	ns	0.02	ns	−0.03	ns	0.02	ns
GlycA, µmol/L	−0.02	ns	0.06	ns	−0.07	ns	0.10	ns
WBC, ×10^9^/L	−0.13	ns	0.10	ns	0.06	ns	−0.03	ns
PMN, ×10^9^/L	−0.07	ns	0.06	ns	0.05	ns	−0.04	ns
NLR	0.03	ns	0.02	ns	0.02	ns	0.03	ns
MLR	0.13	ns	−0.02	ns	−0.05	ns	−0.07	ns

ApoB and GlycA levels were determined in 104 patients. ns = non-significant; HDL = high-density lipoprotein cholesterol; TG = triglyceride; apo = apolipoprotein; hsCRP = high-sensitivity C-reactive protein; WBC = white blood cell; PMN = polymorphonuclear neutrophil; NLR = neutrophil-to-lymphocyte ratio; MLR = monocyte-to-lymphocyte ratio.

**Table 6:** Analysis of HDL-C, TG, remnant cholesterol and apoB between healthy controls (n = 54) and patients with SZ (n = 147) adjusted for hsCRP and GlycA.

	Healthy Controls (n = 54)	SZ (n = 147)	*p* Value
**Adjusted for hsCRP**			
Log_10_ HDL-C, mmol/L	0.16 ± 0.02	0.05 ± 0.01	<0.001
Log_10_ TG, mmol/L	0.03 ± 0.04	0.23 ± 0.02	<0.001
Log_10_ remnant cholesterol, mmol/L	−0.31 ± 0.04	−0.15 ± 0.02	<0.001
Log_10_ apoB, µmol/L	0.21 ± 0.02	0.21 ± 0.01	ns
**Adjusted for GlycA**			
Log_10_ HDL-C, mmol/L	0.17 ± 0.02	0.05 ± 0.01	<0.001
Log_10_ TG, mmol/L	0.01 ± 0.04	0.23 ± 0.03	<0.001
Log_10_ remnant cholesterol, mmol/L	−0.34 ± 0.04	−0.15 ± 0.03	<0.001
Log_10_ apoB, µmol/L	0.23 ± 0.02	0.20 ± 0.01	ns
**Adjusted for hsCRP and GlycA**			
Log_10_ HDL-C, mmol/L	0.16 ± 0.02	0.05 ± 0.01	<0.001
Log_10_ TG, mmol/L	0.02 ± 0.04	0.23 ± 0.03	<0.001
Log_10_ remnant cholesterol, mmol/L	−0.33 ± 0.04	−0.16 ± 0.03	<0.001
Log_10_ apoB, µmol/L	0.22 ± 0.02	0.20 ± 0.01	ns

Results are expressed as estimated marginal means ± standard error, and non-parametrically distributed variables were log_10_ transformed before analysis. GlycA levels were determined in 104 patients. log_10_ = logarithm base 10; ns = non-significant; SZ = schizophrenia; HDL-C = high-density lipoprotein cholesterol; TG = triglyceride; apo = apolipoprotein; hsCRP = high-sensitivity C-reactive protein.

**Table 7:** Analysis of hsCRP and GlycA between healthy controls (n = 54) and patients with SZ (n = 147) adjusted for HDL-C, TG, remnant cholesterol and apoB.

	Healthy Controls (n = 54)	SZ (n = 147)	*p* Value
**Adjusted for HDL-C**			
Log_10_ hsCRP, mg/L	−0.06 ± 0.07	0.35 ± 0.04	<0.001
Log_10_ GlycA, µmol/L	2.56 ± 0.01	2.65 ± 0.01	<0.001
**Adjusted for TG**			
Log_10_ hsCRP, mg/L	−0.08 ± 0.07	0.36 ± 0.04	<0.001
Log_10_ GlycA, µmol/L	2.56 ± 0.01	2.64 ± 0.01	<0.001
**Adjusted for remnant cholesterol**			
Log_10_ hsCRP, mg/L	−0.10 ± 0.07	0.36 ± 0.04	<0.001
Log_10_ GlycA, µmol/L	2.56 ± 0.01	2.64 ± 0.01	<0.001
**Adjusted for apoB**			
Log_10_ hsCRP, mg/L	−0.12 ± 0.08	0.37 ± 0.05	<0.001
Log_10_ GlycA, µmol/L	2.56 ± 0.01	2.65 ± 0.01	<0.001
**Adjusted for HDL-C, TG, remnant cholesterol and apoB**			
Log_10_ hsCRP, mg/L	−0.07 ± 0.08	0.34 ± 0.05	<0.001
Log_10_ GlycA, µmol/L	2.56 ± 0.01	2.64 ± 0.01	<0.001

Results are expressed as estimated marginal means ± standard error, and non-parametrically distributed variables were log_10_ transformed before analysis. GlycA levels were determined in 104 patients. log_10_ = logarithm base 10; SZ = schizophrenia; HDL-C = high-density lipoprotein cholesterol; TG = triglyceride; apo = apolipoprotein; hsCRP = high-sensitivity C-reactive protein.

**Table 8:** Unadjusted analysis of cytokine markers between healthy controls (n = 19) and patients with SZ (n = 53).

	Healthy Controls (n = 19)	SZ (n = 53)	*p* Value
IL-1β, pg/mL	0.6 (0.1–1.8)	0.8 (0.1–4.6)	ns
IL-6, pg/mL	5.0 (3.4–5.9)	5.9 (4.1–10.5)	ns
TNF-α, pg/mL	6.9 (4.4–8.9)	8.3 (6.6–11.7)	0.05
IFN-γ, pg/mL	3.9 (2.3–12.7)	6.6 (3.6–29.5)	ns
IL-4, pg/mL	12.6 (10.3–19.2)	17.2 (10.1–31.4)	ns
IL-10, pg/mL	1.9 (0.9–5.0)	6.9 (2.1–16.2)	0.004
IL-6/IL-10	2.7 (1.6–5.2)	1.2 (0.5–2.5)	0.01
TNF-α/IL-10	4.7 (2.1–6.5)	1.4 (0.6–3.8)	0.01
IFN-γ/IL-4	0.4 (0.1–0.9)	0.4 (0.2–1.1)	ns
IFN-γ/IL-10	2.4 (1.2–5.6)	1.3 (0.7–3.7)	ns

Cytokine levels were measured using MILLIPLEX MAP Human Cytokine/Chemokine panel kit as described in the method. Results are expressed as median and (interquartile range). ns = non-significant; SZ = schizophrenia; IL = interleukin; TNF = tumour necrosis factor; IFN = interferon.

**Table 9:** Spearman’s rank correlations between lipids and cytokines in patients with schizophrenia (n = 53) in the study cohort subset.

	HDL-C, mmol/L		TG, mmol/L		Remnant Cholesterol, mmol/L		apoB, µmol/L	
	ρ	*p* Value	ρ	*p* Value	ρ	*p* Value	ρ	*p* Value
IL-1β, pg/mL	0.25	ns	−0.24	ns	−0.26	ns	−0.31	0.03
IL-6, pg/mL	0.27	ns	−0.31	0.02	−0.34	0.02	−0.30	0.03
TNF-α, pg/mL	−0.01	ns	−0.02	ns	0.01	ns	0.07	ns
IFN-γ, pg/mL	0.31	0.02	−0.26	ns	−0.26	ns	−0.21	ns
IL-4, pg/mL	0.37	0.01	−0.25	ns	−0.20	ns	−0.33	0.02
IL-10, pg/mL	0.21	ns	−0.21	ns	−0.16	ns	−0.17	ns
IL-6/IL-10	−0.04	ns	0.08	ns	0.07	ns	0.07	ns
TNF-α/IL-10	−0.16	ns	0.22	ns	0.20	ns	0.21	ns
IFN-γ/IL-4	0.20	ns	−0.16	ns	−0.21	ns	−0.05	ns
IFN-γ/IL-10	0.19	ns	−0.08	ns	−0.10	ns	−0.08	ns

ns = non-significant; HDL-C = high-density lipoprotein-cholesterol; TG = triglyceride; apo = apolipoprotein; IL = interleukin; TNF = tumour necrosis factor; IFN = interferon.

## Data Availability

The original contributions presented in this study are included in the article/Supplementary Materials. Jeffrey Wang had full access to all the data in the study and takes responsibility for its integrity and data analysis. Further inquiries can be directed to the corresponding author M.K.

## Supplementary Materials

The following supporting information can be downloaded at: https://www.mdpi.com/article/10.3390/biomedicines12122884/s1, Table S1: Lipid parameters between healthy men and women controls and men and women with SZ. Table S2: hsCRP and GlycA between healthy men and women controls and men and women with SZ. Table S3: Clinical characteristics of the study subset cohort for which cytokine data were available (n = 72). Table S4: Lipid parameters between healthy controls (n = 19) and patients with SZ (n = 53) in the study cohort subset for which cytokine data were available. Table S5: Circulating inflammatory markers between healthy controls (n = 19) and patients with SZ (n = 53) in the study cohort subset for which cytokine data were available. Table S6: Multivariable-adjusted analysis of cytokine markers between healthy controls (n = 19) and patients with SZ (n = 53) in the study cohort subset.
