# Supplementary material for: Discordance Between Triglycerides, Remnant Cholesterol and Systemic Inflammation in Patients with Schizophrenia
Source: Biomedicines. 2024 Dec 18;12(12):2884. doi: 10.3390/biomedicines12122884 (PMC11673878; doi:10.3390/biomedicines12122884)
Supplement: Supplementary file 1 [file biomedicines-12-02884-s001.zip › biomedicines-3366422-supplementary.pdf]

Supplementary Material:

Supplementary Table S1: Lipid parameters between healthy men and women controls and men and women with SZ.

	Men (n = 126)			Women (n = 77)		
	**Healthy controls (n = 29)**	**SZ (n = 97)**	***p* value**	**Healthy controls (n = 27)**	**SZ (n = 50)**	***p* value**
TC, mmol/L	4.9 (4.3-5.6)	4.8 (4.1-5.6)	ns	4.9 (4.4-5.8)	5.1 (4.4-5.7)	ns
LDL-C, mmol/L	2.8 (2.5-3.5)	2.9 (2.2-3.7)	ns	2.5 (2.1-3.6)	3.0 (2.5-3.5)	ns
HDL-C, mmol/L	1.3 (1.1-1.5)	1.0 (0.9-1.2)	<0.001	1.7 (1.4-2.1)	1.4 (1.2-1.4)	0.002
TG, mmol/L	0.9 (0.7-1.6)	1.9 (1.4-2.6)	<0.001	0.9 (0.7-1.1)	1.4 (1.0-2.0)	0.003
Remnant cholesterol, mmol/L	0.4 (0.3-0.7)	0.8 (0.6-1.1)	<0.001	0.4 (0.3-0.5)	0.6 (0.4-0.8)	0.06
apoB, µmol/L	1.7 (1.4-2.0)	1.6 (1.4-2.0)	ns	1.4 (1.3-2.0)	1.6 (1.3-2.0)	ns

Results are expressed as median and (interquartile range). ApoB levels were determined in 89 men and 61 women. ns = non-significant; SZ = schizophrenia; TC = total cholesterol; LDL-C = low-density lipoprotein cholesterol; HDL-C = high-density lipoprotein cholesterol; TG = triglyceride; apo = apolipoprotein.

Supplementary Table S2: hsCRP and GlycA between healthy men and women controls and men and women with SZ.

	Men (n = 126)			Women (n = 77)		
	**Healthy controls (n = 29)**	**SZ (n = 97)**	***p* value**	**Healthy controls (n = 27)**	**SZ (n = 50)**	***p* value**
hsCRP, mg/L	0.8 (0.3-1.3)	2.5 (0.9-5.0)	<0.001	1.0 (0.3-2.1)	2.9 (0.8-7.0)	0.004
GlycA, µmol/L	351.0 (307.8-419.5)	429.0 (394.0-499.0)	<0.001	359.0 (316.3-405.8)	437.0 (402.0-516.0)	<0.001

Results are expressed as median and (interquartile range). GlycA levels were determined in 89 men and 61 women. SZ = schizophrenia; hsCRP = high-sensitivity C-reactive protein.

Supplementary Table S3: Clinical characteristics of the study subset cohort for which cytokine data were available (n = 72).

	**Healthy controls (n = 19)**	**SZ (n = 53)**	***p* value**
Age, years	42.3 ± 12.7	42.1 ± 12.9	ns
Biological sex, M/F; %M	12/7 (63)	35/18 (66)	ns
BMI, kg/m^2	23.4 ± 2.5	30.4 ± 5.6	<0.001
HbA1c, %	5.5 (5.2-5.7)	5.9 (5.4-6.2)	0.02
Smoking, % current	1/19 (5)	19/53 (36)	0.01
Diabetes, %	2/19 (11)	13/53 (25)	ns
Statin therapy, %	0/19 (0)	13/53 (25)	0.02
Hypertension, %	4/19 (21)	7/53 (13)	ns
SZ duration of illness, years	-	15.1 (10.3-24.8)	-
Clozapine therapy, %	-	37/53 (70)	-

Results are expressed as number and (%), mean ± standard deviation, or median and (interquartile range). HbA1c levels were determined in 19 controls and 47 patients. ns = non-significant; SZ = schizophrenia; BMI = body mass index; HbA1c = haemoglobin A1c.

Supplementary Table S4: Lipid parameters between healthy controls (n = 19) and patients with SZ (n = 53) in the study cohort subset for which cytokine data were available.

	Healthy controls (n = 19)	SZ (n = 53)	*p* value
TC, mmol/L	4.9 ± 0.7	5.2 ± 1.1	ns
LDL-C, mmol/L	2.9 (2.5-3.1)	3.2 (2.5-4.0)	ns
HDL-C, mmol/L	1.4 ± 0.3	1.1 ± 0.3	0.005
TG, mmol/L	1.0 (0.7-1.6)	1.6 (1.2-2.4)	0.004
Remnant cholesterol, mmol/L	0.4 (0.3-0.7)	0.7 (0.5-1.0)	0.01
apoB, µmol/L	1.8 ± 0.6	1.8 ± 0.4	ns

Results are expressed as mean ± standard deviation, median and (interquartile range). ApoB levels were determined in 11 controls and 52 patients. ns = non-significant; SZ = schizophrenia; HDL-C = high-density lipoprotein cholesterol; TG = triglyceride; apo = apolipoprotein.

Supplementary Table S5: Circulating inflammatory markers between healthy controls (n = 19) and patients with SZ (n = 53) in the study cohort subset for which cytokine data were available.

	**Healthy controls (n = 19)**	**SZ (n = 53)**	***p* value**
hsCRP, mg/L	0.6 (0.3-1.1)	0.5 (0.3-0.6)	<0.001
GlycA, µmol/L	382.5 ± 81.6	473.5 ± 93.3	0.005
WBC, x10^9/L	6.4 ± 1.5	8.2 ± 2.4	<0.001
PMN, x10^9/L	3.5 ± 1.0	5.2 ± 2.1	<0.001
Monocytes, x10^9/L	0.6 (0.3-0.6)	0.7 (0.5-0.8)	0.01
Lymphocytes, x10^9/L	2.0 (1.8-2.5)	2.1 (1.7-2.7)	ns
NLR	1.6 (1.3-2.0)	2.3 (1.6-3.2)	0.005
MLR	0.2 (0.2-0.3)	0.3 (0.2-0.4)	<0.001

Results are expressed as mean ± standard deviation, or median and (interquartile range). GlycA levels were determined in 11 controls and 52 patients. ns = non-significant; SZ = schizophrenia; hsCRP = high-sensitivity C-reactive protein; WBC = white blood cell; PMN = polymorphonuclear neutrophil; NLR = neutrophil to lymphocyte ratio; MLR = monocyte to lymphocyte ratio.

Supplementary Table S6: Multivariable-adjusted analysis of cytokine markers between healthy controls (n = 19) and patients with SZ (n = 53) in the study cohort subset.

	Healthy controls (n = 19)	SZ (n = 53)	*p* value
Log_{10} IL-1β, pg/mL	-0.51 ± 0.33	-0.11 ± 0.19	ns
Log_{10} IL-6, pg/mL	0.67 ± 0.12	0.89 ± 0.07	ns
Log_{10} TNF-α, pg/mL	0.86 ± 0.10	0.99 ± 0.06	ns
Log_{10} IFN-γ, pg/mL	0.72 ± 0.24	0.99 ± 0.13	ns
Log_{10} IL-4, pg/mL	1.11 ± 0.14	1.30 ± 0.08	ns
Log_{10} IL-10, pg/mL	0.14 ± 0.23	0.73 ± 0.13	0.01
Log_{10} IL-6/IL-10	0.54 ± 0.15	0.15 ± 0.09	0.01
Log_{10} TNF-α/IL-10	0.72 ± 0.20	0.25 ± 0.11	0.01
Log_{10} IFN-γ/IL-4	-0.39 ± 0.16	-0.31 ± 0.09	ns
Log_{10} IFN-γ/IL-10	0.58 ± 0.20	0.26 ± 0.11	ns

Cytokine levels were measured using MILLIPLEX MAP Human Cytokine/Chemokine panel kit as described in the methods. Results are expressed as estimated marginal means ± standard error, and non-parametrically distributed variables were log_{10} transformed before analysis. Multivariable models were adjusted for age, sex, body mass index, diabetes, smoking and statin use. log_{10} = logarithm base 10; ns = non-significant; SZ = schizophrenia; IL = interleukin; TNF = tumour necrosis factor; IFN = interferon.
